# Sparse convolutional neural network for high-resolution skull shape completion and shape super-resolution

**DOI:** 10.1038/s41598-023-47437-6

**Published:** 2023-11-19

**Authors:** Jianning Li, Christina Gsaxner, Antonio Pepe, Dieter Schmalstieg, Jens Kleesiek, Jan Egger

**Affiliations:** 1 Institute for AI in Medicine (IKIM), University Medicine Essen (AöR), Girardetstraße 2, 45131 Essen, Germany; 2 Institute of computer graphics and vision, Graz University of Technology, Graz, Austria

**Keywords:** Biomedical engineering, Fracture repair

## Abstract

Traditional convolutional neural network (CNN) methods rely on dense tensors, which makes them suboptimal for spatially sparse data. In this paper, we propose a CNN model based on sparse tensors for efficient processing of high-resolution shapes represented as binary voxel occupancy grids. In contrast to a dense CNN that takes the entire voxel grid as input, a sparse CNN processes only on the non-empty voxels, thus reducing the memory and computation overhead caused by the sparse input data. We evaluate our method on two clinically relevant skull reconstruction tasks: (1) given a defective skull, reconstruct the complete skull (i.e., skull shape completion), and (2) given a coarse skull, reconstruct a high-resolution skull with fine geometric details (shape super-resolution). Our method outperforms its dense CNN-based counterparts in the skull reconstruction task quantitatively and qualitatively, while requiring substantially less memory for training and inference. We observed that, on the 3D skull data, the overall memory consumption of the sparse CNN grows approximately linearly during inference with respect to the image resolutions. During training, the memory usage remains clearly below increases in image resolution—an $\times 8$ increase in voxel number leads to less than $\times 4$ increase in memory requirements. Our study demonstrates the effectiveness of using a sparse CNN for skull reconstruction tasks, and our findings can be applied to other spatially sparse problems. We prove this by additional experimental results on other sparse medical datasets, like the aorta and the heart. Project page at https://github.com/Jianningli/SparseCNN.

## Introduction

One of the challenges of transferring recent advances in 3D shape analysis to the medical field is that the 3D objects in typical benchmark datasets are of small to moderate sizes. Thus, memory efficiency is often not a primary concern. When applied to medical images, these algorithms often exceed available memory, even on a high-end GPU with many Gigabytes of memory. For example, the 3D models (e.g., chairs, cars, airplanes, etc.) in ShapeNet collection typically consist of a few thousand points, while a typical high-resolution 3D CT scan yields millions of points when converted to a point cloud representation^1^.

An obvious opportunity to address the memory issues lies in exploiting spatial sparsity of the 3D data. Some medical data sets, such as the skull, are inherently sparse, with voxel occupancy rates as low as 10%. Since only non-empty voxels carry geometric information of the 3D shape, a sparse convolutional neural network (CNN)^2–5^ can save both memory and computational effort.

In our work, we construct a sparse CNN using the *Minkowski Engine*^5^, which was originally designed for spatio-temporal tensors of 4D and up. We demonstrate how to apply the same principles to sparse, binary volumetric data. To that aim, we evaluate our sparse CNN on two skull reconstruction tasks: skull shape completion and skull shape super-resolution. With sparse CNN, the skull images can be processed in their original resolution ($512 \times 512 \times Z$, where *Z* is the number of axial slices in a head CT scan) with moderate memory requirement. Results show the superiority of sparse CNN over conventional dense CNN in terms of both runtime performance and memory requirements on sparse data.

This paper is an extension of our submission^6^ to the AutoImplant 2021 challenge (https://autoimplant2021.grand-challenge.org/). Reference^6^ first demonstrated that it is feasible to use sparse CNN in skull reconstruction tasks and empirically analysed its advantages over regular CNN. Compared to Ref.^6^, the major improvements of this work are summarized as follows:

- Only the edges of the skulls were used in Ref.^6^, as the available GPU memory is rather low (6 GB), resulting in suboptimal skull reconstructions. In this work, we can use the whole dense skulls thanks to the extended GPU capacity (12 GB).
- The superiority claim of sparse CNN in Ref.^6^ is substantiated by experimental evidence in our work by comparing the sparse CNN with its dense counterparts regarding reconstruction accuracy and computation efficiency (e.g., memory usage, training speed).
- Besides shape completion, we show in this work that the proposed sparse CNN can also be used for shape super-resolution for a variety of binary voxel grids representations besides the skulls.
- We show that the sparse CNN can be used as an integral component in medical image segmentation tasks to further refine the binary segmentation masks initially produced by a dense CNN-based segmentation network.

## Related work

### Shape completion

 Shape completion refers to the process of restoring the missing regions of an object represented as point clouds^7^, meshes^8^ or voxel grids^9–11^. Due to the regularity of voxel grids, using voxel grids for completion takes the advantage of existing and well-established CNN architectures, such as auto-encoders, which are designed to process *images*. However, an object that is originally acquired in point clouds has to be voxelized to a high-resolution voxel grid in order to preserve its geometric details. Nevertheless, the use of a voxel grid in learning-based approaches is expensive, as memory requirements grow cubically with respect to resolution, often resulting in coarse reconstructions. The work of Han et al.^9^ and Dai et al.^10^ addressed the memory issues of voxel grid completion by reconstructing high resolution voxel grids in a two-step, coarse-to-fine fashion. Other studies work around the memory issue by using only very coarse voxel grids (e.g., $24\times 54\times 24$) for completion^11^.

**Figure 1:**
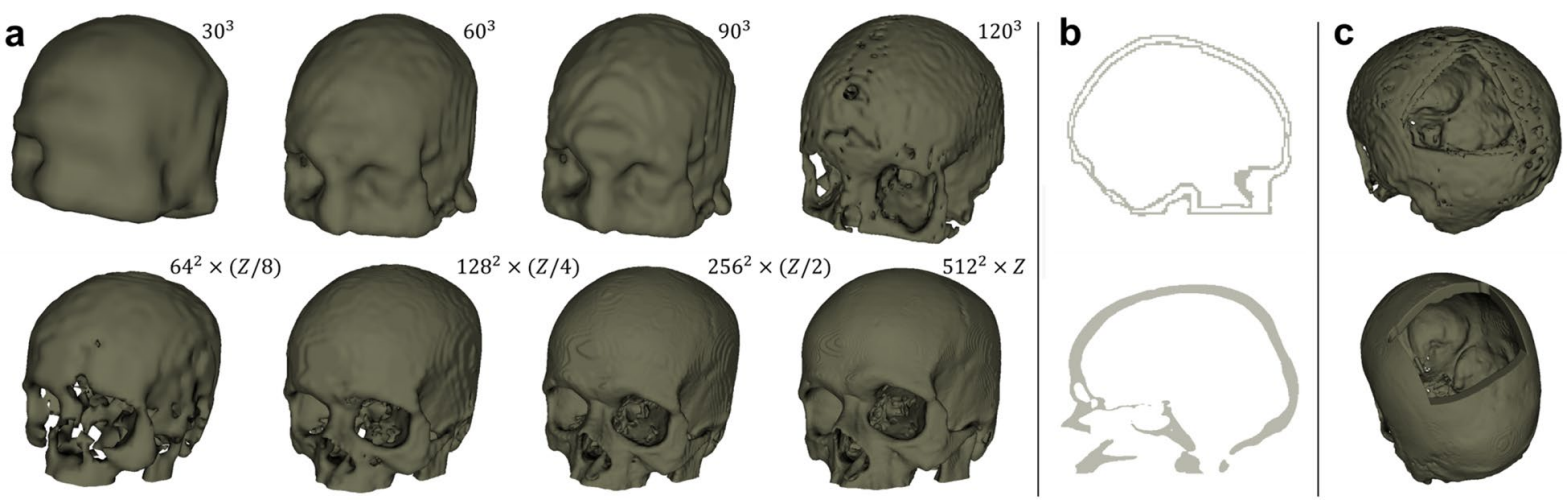
Illustration of the binarized MRI (first row) and CT (second row) skull dataset. (**a**) the MRI and CT skulls at different resolutions. (**b**) midsagittal views of an MRI and CT skull. (**c**) MRI and CT skulls with synthetic defects.

Compared to voxel grids, point clouds are much more light-weight and efficient representations in 3D. Yuan et al.^7^ proposed a deep learning framework that performs shape completion directly on raw point clouds data without voxelization. However, since point clouds are unstructured and objects of the same size differ in their number of points, deep learning has to deal with irregular memory access^12,13^. These CNN methods for shape completion generally used the auto-encoder architecture and its variants.

To further address the memory issues and improve the reconstruction quality, recent arts in learning-based 3D reconstruction propose to represent 3D shapes as implicit functions, such that 3D reconstruction/completion can be learnt directly in the function space^14–16^. Since implicit functions of 3D shapes are not reliant on a specific resolution, 3D shapes can be extracted from the learned implicit functions at arbitrary resolutions, achieving reconstructions in a continuous space. Aside from the memory issues, ill-posedness is another actively studied problem in shape completion, considering that there could exist multiple feasible reconstructions given one incomplete observation. Recent arts propose a shape completion framework based on autoregressive models (e.g., image transformer^17^) that learn a distribution of completions, from which multiple feasible completions respecting the input can be sampled. In Ref.^18^, the authors exploit the advantages of both implicit 3D representations and image transformer to learn high-resolution and varied shape reconstructions from partial observations. In this paper, we focus on solving the memory issues while learning a deterministic mapping between a defective and a complete skull, both represented as a binary voxel grid.

### Skull shape completion and clinical applications

 Skull shape completion has important applications in craniofacial implant design^1,19,20^. The skull images are segmented as binary voxel grids from high-resolution CT scans, typically at a resolution of $512\times 512\times Z$. In CNN applications, the size of such skull images significantly exceeds the memory capacity of a standard desktop GPU. Previous methods either downsample^21^ or resample^22,23^ the skull images to a smaller, intermediate size, or use a patch-wise training and inference strategy^1^. Li et al.^24^ proposed a two-step, coarse-to-fine framework that generates high-resolution implants with reduced memory usage. All these methods are far from optimal, as downsampling or resampling inevitably results in image quality degradation and, consequently, deformation of the skull shape. The two-step method proposed by Li et al.^24^ is not end-to-end trainable in its original form. The patch-based approach requires a tailored training strategy to make sure that the CNN captures the overall shape distribution of the human skull^1^. Besides, it was reported in Ref.^1^ that the reconstructed high-resolution skulls would appear *patchy* due to the incongruency around the borders of the individual patches. Furthermore, Refs.^24,25^ also showed that a network would be more likely to learn the overall shape distributions of the skulls when given an entire skull as input after downsampling, compared to given only a portion (e.g., a bounding box^24^ or a patch^25^) of the skull. The full-image context helps increase a network’s robustness against defect patterns and generalizability. On this account, an ideal CNN for skull reconstruction should take the entire high-resolution skull images as input and output the reconstructed skulls or implants in their original resolutions.

### Data spatial sparsity and sparse CNN

 In a recent approach^25^, the authors adopted a hash table to exploit the sparse and binary structure of the skull images to reduce the reconstruction time and memory consumption. Instead of the entire skull volume, the method reconstructs only the non-zero voxels and stores them as bit-strings, so that each voxel occupies only one bit of memory. This is a non-CNN approach and requires that voxel coordinates are stored during reconstruction to maintain the spatial relationship among the reconstructed voxels.

In this paper, we propose to take the advantage of such spatial sparsity of the skull data to reduce memory consumption using only sparse convolutions. Note that by “sparse CNN” we mean a CNN architecture made for sparse input data (like the skull) and not a compressed CNN with sparse (e.g., mostly zero) parameters^26–29^. This makes our approach conceptually similar to methods which apply a CNN to 3D shapes at high resolutions, such as Riegler et al.^3^ and Wang et al.^2^. Both used an octree representation for 3D shapes and proposed octree-based convolutions. Graham et al.^4,30^ and Choy et al.^5^ proposed sparse convolutions defined on the non-empty points in an object. During execution, features are extracted only from these non-empty locations, such that the zero-valued background does not take up memory and computation resources.

## Data generation

We used two public skull datasets in our study, namely, the MRI skull dataset from the Human Connectome Project (HCP, https://humanconnectome.org/study/hcp-young-adult/document/1200-subjects-data-release/) and the CT skull dataset from Task 3 of the AutoImplant 2021 challenge.

**Figure 2:**
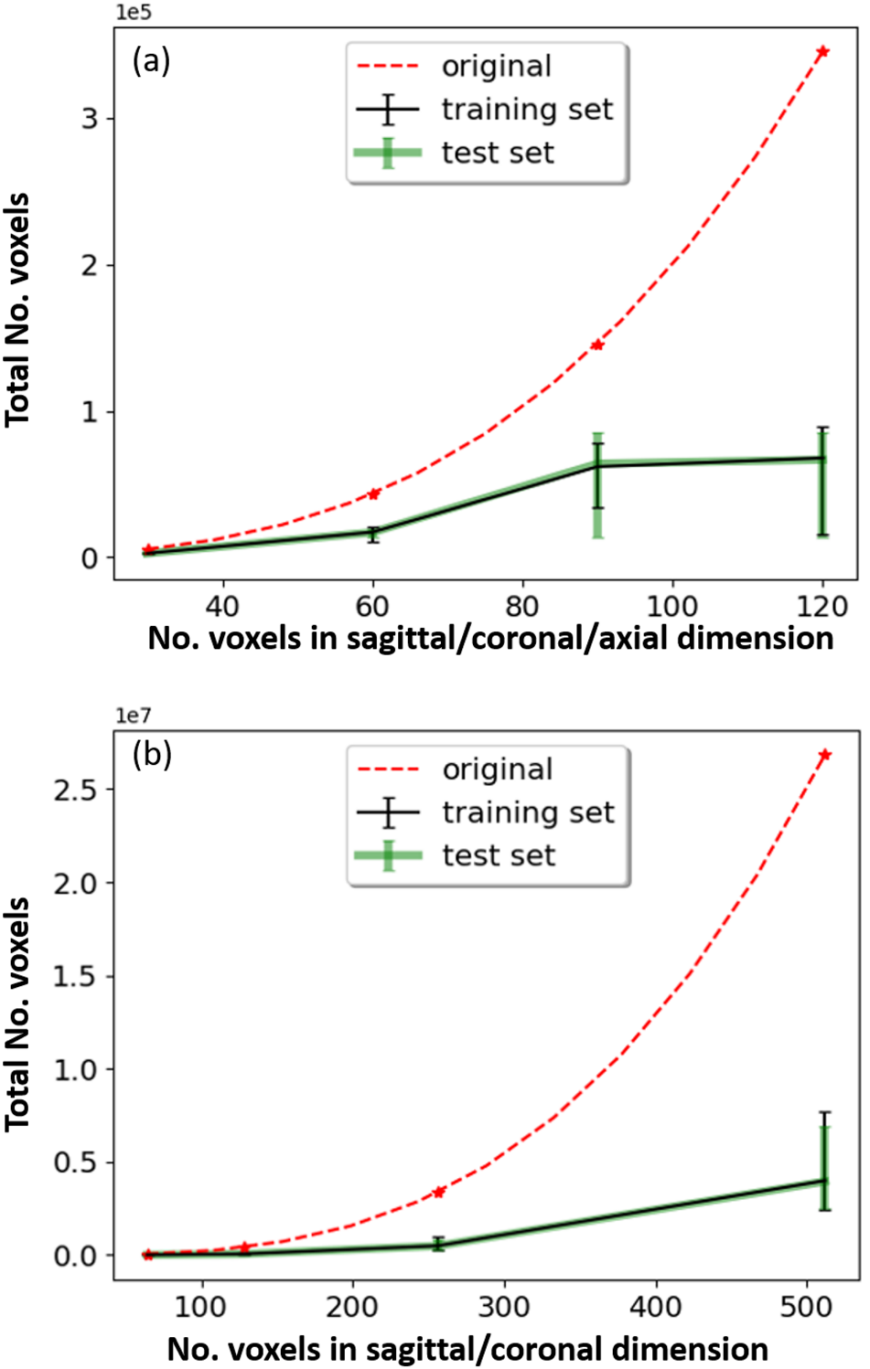
Memory occupancy (as shown in the vertical axis, which is represented as the total number of relevant voxels) of the MRI (**a**) and CT (**b**) skull datasets at different resolutions (as shown in the horizontal axis, which is represented as the number of voxels in the sagittal, coronal and/or axial image dimensions). For the *original* skull data, the memory occupancy was down-scaled by a factor of ten and five for the MRI and CT dataset, respectively, for the plots. For the CT dataset (**b**), the axial dimension differs for different CT images. The plots in black and green depict the mean, (mean-min) and (max-mean) of the number of voxels.

### MRI dataset

 The HCP dataset originally contains 1113 structural MRI scans, and 200 of them were selected in our study (100 for training and 100 for evaluation). The BrainSuite (http://brainsuite.org/) software was used to extract the skull surfaces from the scans^31^. Note that the program extracts only the interior and exterior skull surfaces as can be seen from Fig. 1b. The skull meshes were further voxelized to binary grid representations at various resolutions: $30^3$, $60^3$, $90^3$ and $120^3$ ( Fig. 1a).

### CT dataset

 In CT scans, bone structures can be distinguished based on gray values, and therefore the skulls can simply be extracted using thresholding, resulting in binary voxel grids of resolution $512 \times 512 \times Z$ (for all CT images, the *X*, *Y* resolutions are both 512, while the *Z* resolution varies across scans). The dataset contains 100 skulls for training and 100 for evaluation (the 10 out-of-distribution test cases are not included here). We also created the multi-resolution representation of the CT skulls at $64 \times 64 \times (Z/8)$, $128 \times 128 \times (Z/4)$ and $256 \times 256 \times (Z/2)$, as illustrated in Fig. 1a.

For both datasets, a portion of the skull bone (around the cranium area) was removed to simulate the surgical procedure of craniotomy for the experiments on skull shape completion (Fig. 1c). Figure 2 shows a comparison of the memory occupancy between the original skull voxel grids and the non-zero voxels, for the MRI (Fig. 2a) and CT dataset (Fig. 2b) dataset at various resolutions specified above. Note that the plots use the number of voxels to represent the overall memory occupancy directly, as each voxel occupies a constant space. The MRI dataset was stored as *int8* and the CT dataset was stored as *int32*. The plots show that the memory usage of the original skull data grows cubically with respect to image resolutions, while for the valid voxels, memory usage exhibits approximate linear growth in comparison. Intuitively, a sparse CNN relying only on the valid voxels would be more efficient in terms of memory and computation than a dense CNN that takes the entire voxel grids as input.

## Methods

We use the *Minkowski Engine* proposed by Choy et al.^5^ as the backbone of a sparse CNN. *Minkowski Engine* is originally designed as a general-purpose tool for the analysis of 4D spatio-temporal data and uses sparse tensors as the basic data structure. A sparse tensor $\mathscr {F}$ is a generalized representation of a sparse matrix in which most of the points are empty (zero). A third order sparse tensor can be expressed as:

1 $$\begin{aligned} \mathscr {F}(x_i,y_i,z_i)=\left\{ \begin{matrix} f_i, &{} (x_i,y_i,z_i) \in \mathcal {C}\\ 0, &{} otherwise \end{matrix}\right. \end{aligned}$$

where $\mathcal {C}$ is the coordinate matrix (row-wise concatenation of coordinates) of the non-empty points and $f_i \in \mathbb {R}^{N_F}$ is the non-empty value at coordinate $(x_i,y_i,z_i)$. ${N_F}$ is the number of channels at this point. $$\mathcal {F}=\begin{Bmatrix} f_1,f_2,...,f_i,f_{i+1}...\end{Bmatrix}$$ is the feature vector. Sparse CNN relies only on $\mathcal {C}$ and $\mathcal {F}$ for feature computation. In our study, we use sparse CNN specifically on sparse binary volumes of static data, i.e., the skull images, which are typical examples of sparse tensors, since the majority of voxels in a skull image are zero. The input of the sparse CNN consists of a coordinate matrix $\mathcal {C}_{in}$ and the associated feature vectors $\mathcal {F}_{in}$:

2 $$\begin{aligned} \mathcal {C}_{in}=\begin{bmatrix} x_{1}&{} y_{1}&{} z_{1}\\ x_{2}&{} y_{2}&{} z_{2} \\ ...&{} ...&{}...\\ x_{N}&{} y_{N}&{} z_{N} \end{bmatrix} , \mathcal {F}_{in}=\begin{bmatrix} 1\\ 1\\ ...\\ 1 \end{bmatrix} \end{aligned}$$

Here, *N* is the number of non-zero voxels in a skull image. Note that the coordinates we used in our study refer to voxel grid coordinates (e.g., from [0, 0, 0] to [512, 512, *Z*]) instead of the world coordinates of point clouds. Since the skull data are binary, and the number of channels per voxel is one (${N_F}=1$), the feature vector has a format of $\mathcal {F}_{in} \in \mathbb {R}^{N\times 1}$, and the elements in $\mathcal {F}_{in}$ are all 1. For three-dimensional voxel grid coordinates, $\mathcal {C}_{in} \in \mathbb {Z}^{N\times 3}$. A general data pre-processing step for using the sparse CNN is to format the input and ground truth skull images according to Eq. (2).

Similar to existing CNN methods for shape completion, we use an auto-encoder architecture for the task, but we replaced the conventional dense convolutional layers with sparse convolutional layers^5^. Table 1 shows the configuration of each layer in the sparse CNN used for experiments. *ch* is a list of the channel numbers for each layer. As the number of output channels ($C^{out}$) in each layer is no longer constant 1 as in the input skull image, we use $\mathcal {F}_{in}^i \in \mathbb {R}^{C_{i-1}^{out}}$ as a general notation for the output (i.e., the feature vector) of the intermediate layer *i*. The convolution operation at a coordinate $D \in \mathbb {Z}^3$ in the sparse CNN can therefore be defined similar to that of the traditional dense CNN:

3 $$\begin{aligned} \mathcal {F}_{in}^{i+1}(D')=\sum w^i\mathcal {F}_{in}^i(D)+b_i, \end{aligned}$$

where $w^i \in \mathbb {R}^{ C_{i}^{out} \times C_{i-1}^{out}}$ and $b_i$ is the weight matrix and bias of intermediate layer *i*. $D' \in \mathbb {Z}^3$ is the corresponding coordinate in layer $i+1$ mapped from *D*. Note that, unlike a traditional dense CNN that operates on regular voxel grids sequentially, a sparse CNN requires specifying a coordinate mapping in order to know how *D* is mapped to $D'$, as the non-zero voxels can be distributed arbitrarily, and, by extracting only the non-zero voxels, the spatial context within an image is lost. For such coordinate mapping, *Minkowski Engine* uses a pair of voxel indices from the input and ground truth images to memorize the mapping relationship as in a regular voxel grid, leading to coordinates-related computation overhead, comparable to Li et al.^25^. In *Minkowski Engine*, the coordinates and the voxel indices were stored in a hash table (the hash function used is *FNV64-1A*), where the coordinates were used as hash keys to retrieve the original voxel indices of the associated elements in a feature vector. Even if the hash table is not directly involved in feature computation, they determine how an element from the input feature vector is mapped to an element computed according to Eq. (3) in the output feature vector.

### Shape completion

For skull shape completion, the input is a defective skull and the output (ground truth) is the complete skull. It can be divided into two sub-tasks: reconstructing the original defective skull $$\begin{Bmatrix} \mathcal {C}_{in}, \mathcal {F}_{in} \end{Bmatrix}$$ and restoring the missing skull bone (i.e., the implant) $$\begin{Bmatrix} \mathcal {C}_{imp}, \mathcal {F}_{imp} \end{Bmatrix}$$:

4 $$\begin{aligned} \mathcal {C}_{out}^{sc}=\begin{bmatrix} \mathcal {C}_{in}\\ \mathcal {C}_{imp} \end{bmatrix} , \mathcal {F}_{out}^{sc}=\begin{bmatrix} \mathcal {F}_{in}\\ \mathcal {F}_{imp}\ \end{bmatrix} \end{aligned}$$

where

5 $$\begin{aligned} \mathcal {C}_{imp}=\begin{bmatrix} x_{N+1} &{} y_{N+1} &{}z_{N+1} \\ x_{N+2} &{} y_{N+2} &{}z_{N+2} \\ ...&{}... &{}... \\ x_{N+M} &{} y_{N+M} &{}z_{N+M} \end{bmatrix}, \mathcal {F}_{imp}=\begin{bmatrix} 1\\ 1\\ ...\\ 1 \end{bmatrix} \end{aligned}$$

*M* is a variable denoting the number of non-zero voxels in the generated set of coordinates $\mathcal {C}_{imp}$. $\mathcal {F}_{imp} \in \mathbb {R}^{M\times 1}$, and the elements in $\mathcal {F}_{imp}$ are all 1. Obviously, *M* can be different for different skull instances considering the varaitions of skulls and defects. According to Eq. (4), the sparse CNN needs to generate new sets of coordinates $\mathcal {C}_{imp}$ at which the values are non-zero, for the skull shape completion task ($M\ne 0$ such that $\mathcal {C}_{in}\subset \mathcal {C}_{out}^{sc}$). The generative sparse tensor decoder in Table 1 are composed of generative transposed convolutional layers^32^ that are capable of generating new non-zero points absent in the input. Given a sparse tensor $\mathscr {F}$ as input, the output of a transposed convolution $\mathscr {F}'$ can be written as:

6 $$\begin{aligned} \mathscr {F}'[x,y,z]=\sum _{i,j,k \in \mathcal {N}(x,y,z)}\mathcal {W}[x-i,y-j,z-k]\mathscr {F}[i,j,k] \end{aligned}$$

where $(x,y,z) \in \mathcal {C}'$ and $(i,j,k) \in \mathcal {C}$. $\mathcal {W}$ is the kernel weight. $\mathcal {C}$ and $\mathcal {C}'$ are the input and output coordinate matrix respectively, and they have the following relationship:

7 $$\begin{aligned} \mathcal {C}' =\mathcal {C}\otimes [-\frac{Ks-1}{2},...,\frac{Ks-1}{2}]^3 \end{aligned}$$

$\otimes$ denotes outer-product. A point generated by a transposed convolution (*x*, *y*, *z*) has the following constraint with the input coordinate *i*, *j* and *k* according to Eq. (6):

8 $$\begin{aligned} \mathcal {N}(x,y,z)= \begin{Bmatrix} (i,j,k)||x-j|\leqslant \frac{Ks-1}{2},|y-j|\leqslant \frac{Ks-1}{2},|z-j|\leqslant \frac{Ks-1}{2} \end{Bmatrix} \end{aligned}$$

We can see from Eqs. (6), (7) and (8) that using a kernel size greater than two would expand the span (e.g., $[-Ks, Ks]$) of the input coordinates, allowing a transposed convolution to dynamically generate new non-zero points for generative tasks like shape completion. In our specific task, the generative sparse tensor decoder in Table 1 is trained to generate *M* new points, while maintaining the original input coordinates.

Each transposed convolution layer in Table 1 is followed by a *pruning* layer that prunes out undesirable new points, which is essential for maintaining a low memory and computation cost during the generative process. During training, the ground truth masks teach the network when to keep or prune a point. During inference, the ground truth masks are unavailable. The network prunes a point if its feature value is lower than a pre-defined threshold $\tau$. In our network, we choose $\tau =0$.

**Table 1:** Configuration (number of input channels $C^{in}$, output channels $C^{out}$ and kernel size Ks) of each layer in the encoder and decoder of the sparse CNN.

Encoder			Decoder		
$C^{in}$	$C^{out}$	Ks	$C^{in}$	$C^{out}$	Ks
1	ch[0]	3	*ch[6]	**ch[5]**	**4**
ch[0]	ch[1]	2	ch[5]	ch[5]	3
ch[1]	ch[1]	3	*ch[5]	**ch[4]**	**2**
ch[1]	ch[2]	2	ch[4]	ch[4]	3
ch[2]	ch[2]	3	*ch[4]	**ch[3]**	**2**
ch[2]	ch[3]	2	ch[3]	ch[3]	3
ch[3]	ch[3]	3	*ch[3]	**ch[2]**	**2**
ch[3]	ch[4]	2	ch[2]	ch[2]	3
ch[4]	ch[4]	3	*ch[2]	**ch[1]**	**2**
ch[4]	ch[5]	2	ch[1]	ch[1]	3
ch[5]	ch[5]	3	*ch[1]	**ch[0]**	**2**
ch[5]	ch[6]	2	ch[0]	ch[0]	3
ch[6]	ch[6]	3	ch[0]	1	1
–	–	–	Sigmoid		

The generative transposed convolutional layers are marked bold.

Layers with stride 2 are marked with *.

### Shape super-resolution

Skull shape super-resolution refers to the process of transforming a (completed) coarse binary skull shape to its smooth high-resolution representation with fine geometric details. The input is the completed skull at a low resolution $\mathbb {Z}^a$, and the output is the same completed skull at a higher resolution $\mathbb {Z}^b$, $a<b$. Note that for the skull super-resolution task, the coarse and high-resolution skull (i.e., the ground truth) have to be in the same coordinate system for the coordinate mapping in the sparse CNN to work properly, meaning that the coarse skull image needs to be up-scaled to the same size as the target high-resolution image, i.e., $a=b$. The network would fail to converge when, for example, the input is of resolution $64\times 64\times (Z/8)$, while the ground truth is of resolution $256\times 256\times (Z/2)$. By $a=b$ we do not mean that the input and the ground truth have the same *resolution* from the perspective of image quality. Rather, we mean that the input is interpolated to the same size as the ground truth. The up-scaled input still appears blurry and coarse, and lacks geometric details.

According to Ref.^25^, the difference between a (up-scaled) coarse skull voxel grid and a high-resolution voxel grid is simply the arrangement patterns of the zero and non-zero voxels, and, by rearranging the voxels, a coarse skull shape can be upgraded to the high-resolution representation. The total number of non-zero voxels between the two types of skulls shows no statistical differences. Therefore, we use the following to represent the ground truth coordinate matrix $\mathcal {C}_{out}^{sr}$ and feature vector $\mathcal {F}_{out}^{sr}$ for the super-resolution task:

9 $$\begin{aligned} \mathcal {C}_{out}^{sr}=\begin{bmatrix} x_{1} &{} y_{1} &{}z_{1} \\ x_{2} &{} y_{2} &{}z_{2} \\ ...&{}... &{}... \\ x_{N_0} &{} y_{N_0} &{}z_{N_0} \\ ...&{}... &{}... \\ x_{N}' &{} y_{N}' &{}z_{N}' \end{bmatrix} , \mathcal {F}_{out}^{sr}=\begin{bmatrix} 1\\ 1\\ ...\\ 1\\ ...\\ 1 \end{bmatrix}. \end{aligned}$$

If we assume that an up-scaled coarse skull and the ground truth each possesses $N_{sr}$ (a variable) non-zero points and they share $N_0$ (a variable) common points ($N_0 < N_{sr}$), then $N_{sr}-N_0$ non-zero points in the input need to be pruned while $N_{sr}-N_0$ new non-zero points need to be generated in new coordinates. Therefore, the sparse CNN specified in Table 1 is still applicable to the super-resolution task.

### Memory usage and computation complexity

The memory consumption of a neural network comes primarily from the following sources during training time: (1) input and ground truth image batches, (2) the output of the intermediate layers (forward pass), (3) network parameters, (4) memory usage from back-propagation (errors and gradients at each parameter) and (5) optimizers. In test time, the parameters of the network, input image batches and intermediate layers’ output are the main sources of memory usage. In our study, we compare the memory consumption of sparse and dense CNN when the networks have the same configurations (Table 1) and number of parameters $N_{param}$. For both dense and sparse CNN configurations, $N_{param}$ can be estimated as:

10 $$\begin{aligned} N_{param}=\sum _{i} \mathcal {C}^{out}_i\times \mathcal {C}^{in}_i\times Ks^3+\mathcal {C}^{out}_i. \end{aligned}$$

The number of bias in layer *i* is the same as the number of output channels of the layer $\mathcal {C}^{out}_i$. Assuming that the parameters are stored as *float32* (32-bit), sparse and dense CNN consume the same amount of memory in storing these parameters. However, the input and ground truth for a dense CNN are the original voxel grids, while, for a sparse CNN, only the valid non-zero voxels are required, and thus a sparse CNN consumes significantly less memory than dense CNN in loading the input and ground truth image batches, as shown in Fig. 2. Similarly, the size of the output $N_{f^i}$ corresponding to intermediate layers *i* is linear to the feature dimension of the $(i-1)th$ layer $N_{f^{i-1}}$ and is calculated as:

11 $$\begin{aligned} N_{f^i}=\frac{1}{s}(N_{f^{i-1}}+2p-Ks), \end{aligned}$$

where *p* and *s* are the padding and stride size, respectively. According to Eq. (11), the memory consumption of the intermediate layers’ output is also linear to the input image size (Fig. 2).

The memory consumption related to back-propagation and optimizer is tricky to calculate. In our study, we estimate the overall GPU memory usage during training using the *nvidia-smi* command provided by NVIDIA. We query the system GPU memory usage at 50-millisecond intervals for $N_{train}$ training iterations ($N_{train}$ is the number of training samples, and the batch size was set to 1) and take the average of all the queried values as the final amount of memory consumed for training, considering that the number of non-zero voxels are different for each training sample. The static memory occupancy that is not caused by training the network was subtracted from the measurement. For inference, we used the same method except that the measurement was taken when the network loaded the trained parameters and was run on the test set.

Floating points operations (FLOPS) is commonly used to measure computational complexity of a CNN. The FLOPS consumed in CNN layer *i* is the product of $N_{f^i}$, *Ks* and $\mathcal {C}^{out}_i\times \mathcal {C}^{in}_i$. Given the same network configurations ($\mathcal {C}^{out}_i$, $\mathcal {C}^{in}_i$, *Ks*), the FLOPS are linear to $N_{f^i}$ and thus to the input image size (Fig. 2). The sparse CNN therefore is significantly faster than a dense CNN in both training and inference time under the same configurations.

**Figure 3:**
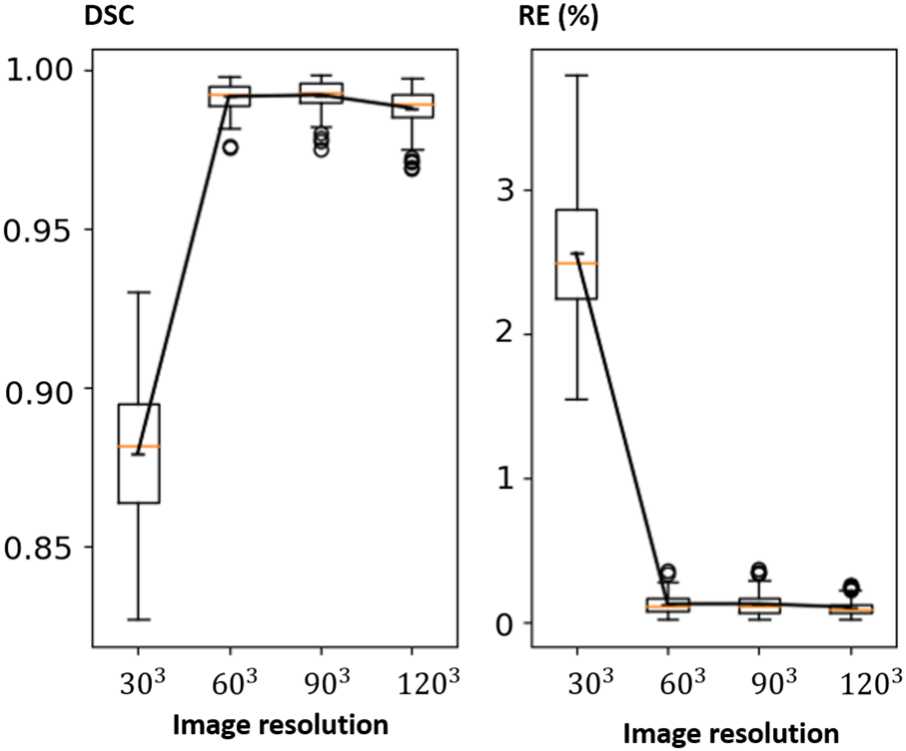
DSC (left) and RE (right, %) for the sparse CNN on the MRI dataset at different resolutions ($30^3$, $60^3$, $90^3$, $120^3$) for the shape completion task. Horizontal axis corresponds to the image resolutions.

**Figure 4:**
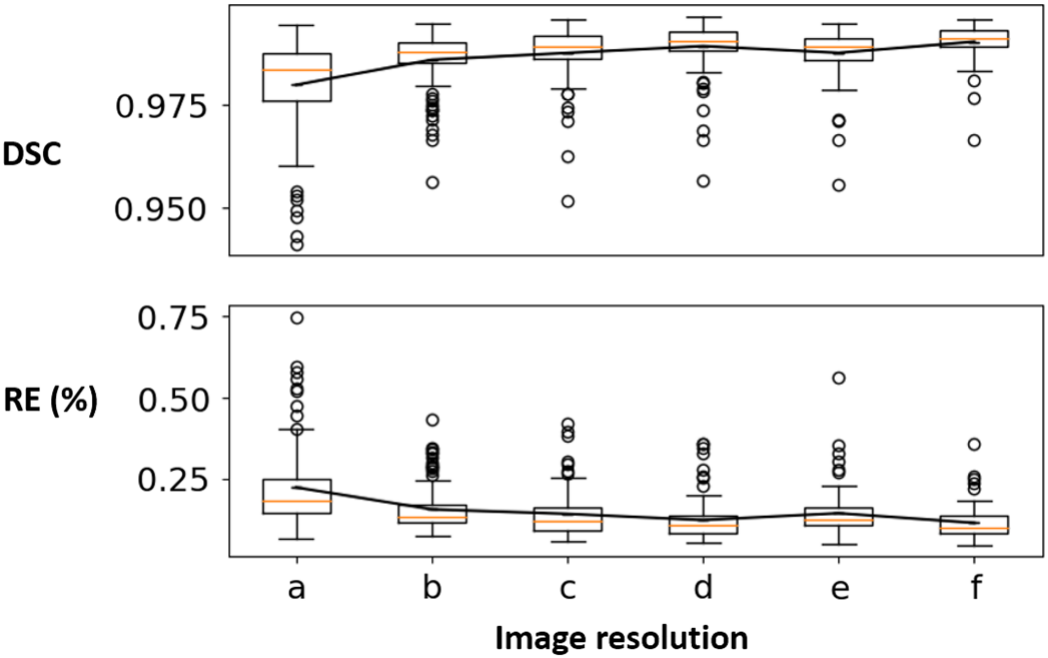
DSC (top) and RE (bottom, %) for the sparse CNN on the CT dataset at different resolutions for the shape completion task. Horizontal axis corresponds to the image resolutions. a: $64^2 \times (Z/8)$ (*ch*1) b: $64^2 \times (Z/8)$ (*ch*2) c: $128^2 \times (Z/4)$ (*ch*1) d: $128^2 \times (Z/4)$ (*ch*2) e: $256^2 \times (Z/2)$ f: $512^2 \times Z$.

## Experiments and results

We trained the sparse CNN (Table 1) for two tasks: The first task is skull shape completion on the CT and MRI skull dataset at different resolutions ($30^3$, $60^3$, $90^3$ and $120^3$ for the MRI dataset and $64^2 \times (Z/8)$, $128^2 \times (Z/4)$, $256^2 \times (Z/2)$ and $512^2 \times Z$ for the CT dataset). For the CT dataset, *ch* is set to $ch1=$ [8, 8, 16, 16, 32, 32, 64] (0.435M parameters), for the MRI dataset, *ch* is set to $ch2=$ [22, 32, 32, 128, 156, 256, 388] (about 18.14M parameters). The second task is skull shape super-resolution on the CT skull dataset on different scales: $64^2 \times (Z/8) \rightarrow 128^2 \times (Z/4)$, $64^2 \times (Z/8) \rightarrow 256^2 \times (Z/2)$, $64^2 \times (Z/8) \rightarrow 512^2 \times Z$ and $128^2 \times (Z/4) \rightarrow 256^2 \times (Z/2)$. It is important to emphasize that, for the CT data, the multi-resolution skull representations are created by downsampling the original skulls (represented as voxel grids) from $512^2 \times Z$ to lower resolutions, whereas for the MRI data, the skulls (represented as meshes) are voxelized to voxel grids at different resolutions. For comparison, we also trained a standard dense CNN with the same configuration as the sparse CNN for the shape completion task on the CT dataset. For both tasks, we used Dice similarity coefficient (DSC) and reconstruction error (RE), i.e., the percentage of misclassified voxels, to evaluate the predictions. The sparse CNN was trained using a binary cross-entropy loss $\mathcal {L}_{bce}$:

12 $$\begin{aligned} \mathcal {L}_{bce}=y'\cdot log\sigma (y)+(1-y')\cdot log(1-\sigma (y)), \end{aligned}$$

and the dense CNN was trained using a Dice loss $\mathcal {L}_{dice}$ for the background ($i=0$) and the target ($i=1$):

13 $$\begin{aligned} \mathcal {L}_{dice}=-2\sum _{i=0}^{1}\frac{\sum y^{i}\circ y'^{i}}{\sum y^{i}\circ y^{i}+\sum y'^{i}\circ y'^{i}}, \end{aligned}$$

$\sigma$ is a sigmoid non-linearity. *y* and $y'$ denote the predictions and the ground truth, respectively. $\circ$ denotes element-wise multiplication between two matrices. Dice loss is the ideal choice for measuring the differences between two masks. Note that, in skull reconstruction tasks, Dice loss is the de facto loss function for dense CNNs, which output masks^19^. However, a sparse CNN outputs 3D points (e.g., coordinates of voxels grids or point clouds) and cross-entropy loss is the de facto choice.

Table 2 shows the quantitative evaluation results (mean DSC and RE) for the shape completion task. In Table 2, we also reported a performance comparison of the sparse CNN with different numbers of parameters (i.e., *ch*1, *ch*2) at resolutions $64^2 \times (Z/8)$ and $128^2 \times (Z/4)$. Results indicate that increasing the model complexity of the sparse CNN would also lead to increased prediction accuracy, a phenomenon well observed in traditional dense CNN models. It is worth noting that, by using a sparse CNN, we are able to train on the CT skull images at their full resolutions ($512^2 \times Z$) and the results are promising with over 0.99 DSC and less than 0.12% reconstruction error (e.g., in a $512^2 \times 256$ image, only 76772 voxels are misclassified on average). Learning at full resolutions is advantageous compared to learning from downsampled data, where the quality of the input and ground truth is inevitably compromised. It is worth mentioning that the sparse CNN tends to achieve higher DSC at higher resolutions according to the results, and that the highest DSC is achieved at the full resolution ($512^2 \times Z$) using a more lightweight network (*ch*1). In contrast, GPU memory restrictions made training on the $512^2 \times Z$ image resolution using a dense CNN unsuccessful. Furthermore, the quantitative results of the dense CNN were significantly worse than the sparse CNN, as can be seen in Table 2. Note that the shape completion results in Table 2 are not directly comparable to the AutoImplant challenge results for three reasons: (1) For a fair comparison, the dense CNN used the same vanilla network configuration as the sparse CNN, while the challenge submissions used more complex (and different) dense network architectures combined with tailored pre- and post-processing (e.g., data augmentation) to achieve the results^19^. (2) Table 2 reported the results at resolutions $64^2 \times (Z/8)$, $128^2 \times (Z/4)$ and $256^2 \times (Z/2)$ for the dense CNN, while the challenge reported the results at resolution $512^2 \times Z$, i.e., the challenge submissions up-scaled the coarse output (the resolution of which differs for different submissions) to $512^2 \times Z$ before calculating the metrics against the ground truth. (3) The results reported in Table 2 apply to the skulls while the challenge results apply to the implants obtained by taking the difference between the reconstructed and ground truth skulls^19^. Note that, we force the dense CNN to follow a architecture specifically designed for sparse CNNs (Table 1), rather than the other way around, which might lead to a unfair ‘competition’ between the two. Therefore, Table 2 is solely to show a comparison of sparse and dense CNN under one vanilla setting, and the results produced in this specific setting are not generalizable to other dense CNNs in general. To provide an external comparison for the proposed sparse CNN, we refer to^25^, in which a dense network with over 82M parameters was trained on the same CT dataset for skull shape completion. DSC from three variants of the method was reported: 0.7547 for interpolation, 0.7529 for voxel rearrangement and 0.8587 for patch-based training and inference. Quantitatively, the sparse CNN with only 0.435M parameters performs significantly better (DSC of 0.9903). Figures 3 and 4 show the DSC and RE distributions over the test sets on the MRI and CT skull datasets, respectively. To provide a quantitative comparison between the sparse CNN and top-ranked dense CNNs from the AutoImplant challenge^35^, we first extracted the implants by subtracting the defective input from the completed skulls reconstructed by the sparse CNN at resolution $512^2\times Z$, and then calculated DSC, border DSC (bDSC), and 95th percentile Hausdorff Distance (HD95) against the original ground truth implants from the challenge. The results are reported in Table 3. It is worth highlighting that the sparse CNN surpassed the best performing dense CNN^33^ from the challenge in terms of border DSC - an important indicator of the clinical applicability of the implants^35^, despite being significantly more lightweight. A quantitative comparison with our previous work^6^ is also given in Table 3. Note that^6^ and our current work used the same sparse CNN configurations but different formats of the training data. In Ref.^6^, only the boundary of the skulls were used for training due to hardware limitations, while the current work used the original full skull voxel grids. Nevertheless, our previous work^6^ still achieved comparable bDSC to the state-of-the-art dense CNNs without resorting to complex post-processing and intensive data augmentation as in Ref.^33^.

**Table 2:** Quantitative results—DSC and RE for the skull shape completion task on the MRI and CT datasets at different resolutions.

MRI				CT (sparse)						CT (dense)		
30	60	90	120	64(*ch*1)	64(*ch*2)	128 (*ch*1)	128 (*ch*2)	256	512	64	128	256
0.8794	0.9915	**0.9920**	0.9879	0.9798	0.9859	0.9876	0.9892	0.9876	**0.9903**	0.4801	0.4928	**0.6069**
2.5593	0.1324	0.1333	**0.1095**	0.2237	0.1561	0.1423	0.1229	0.1443	**0.1144**	7.9719	6.0845	**4.8014**

The second row shows the image resolutions at which DSC (third row) and RE (%, last row) are calculated.

Significant values are in bold.

**Table 3:** Quantitative Comparison Between the Sparse CNN and the Top-ranked Dense CNNs from the AutoImplant Challenge Regarding Implant Generation at Resolution $512^2\times Z$, in terms of DSC, border DSC (bDSC), and 95th percentile Hausdorff Distance (HD95).

Methods $\backslash$ metrics	DSC	bDSC	HD95
sparse CNN (current work)	0.8837	**0.9566**	4.105
sparse CNN^6^	0.8544	0.9463	2.6457
Wodzinski et al.^33^	**0.9336**	0.9530	**1.4761**
Mahdi et al.^34^	0.9154	0.9512	1.6848

Significant values are in bold.

**Figure 5:**
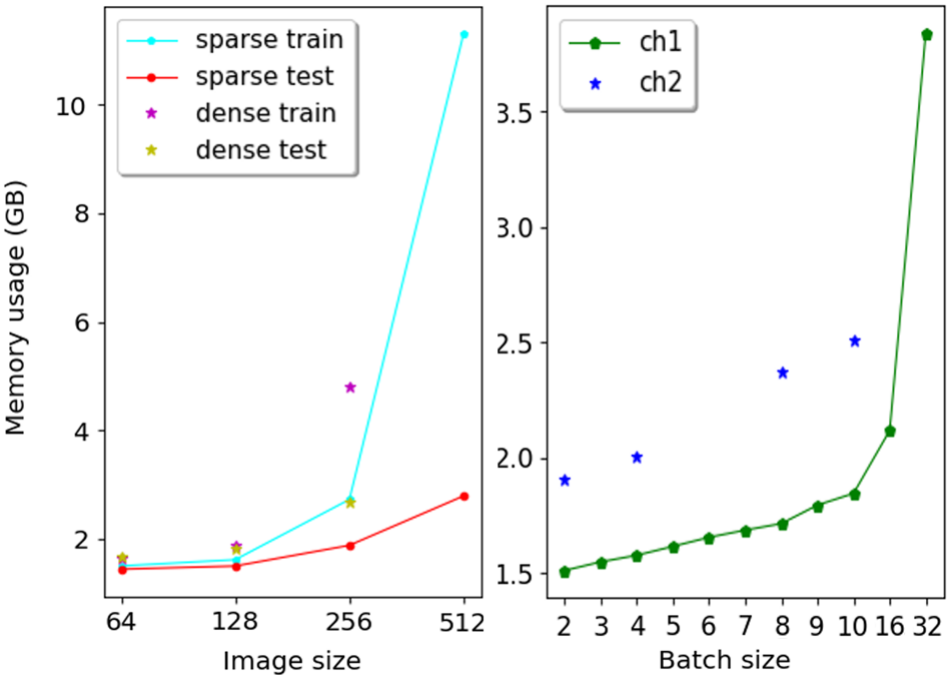
Memory consumption during training and inference for the sparse and dense CNN at different resolutions (left). Memory consumption of sparse CNN with different batch sizes at resolution 64 (right).

**Figure 6:**
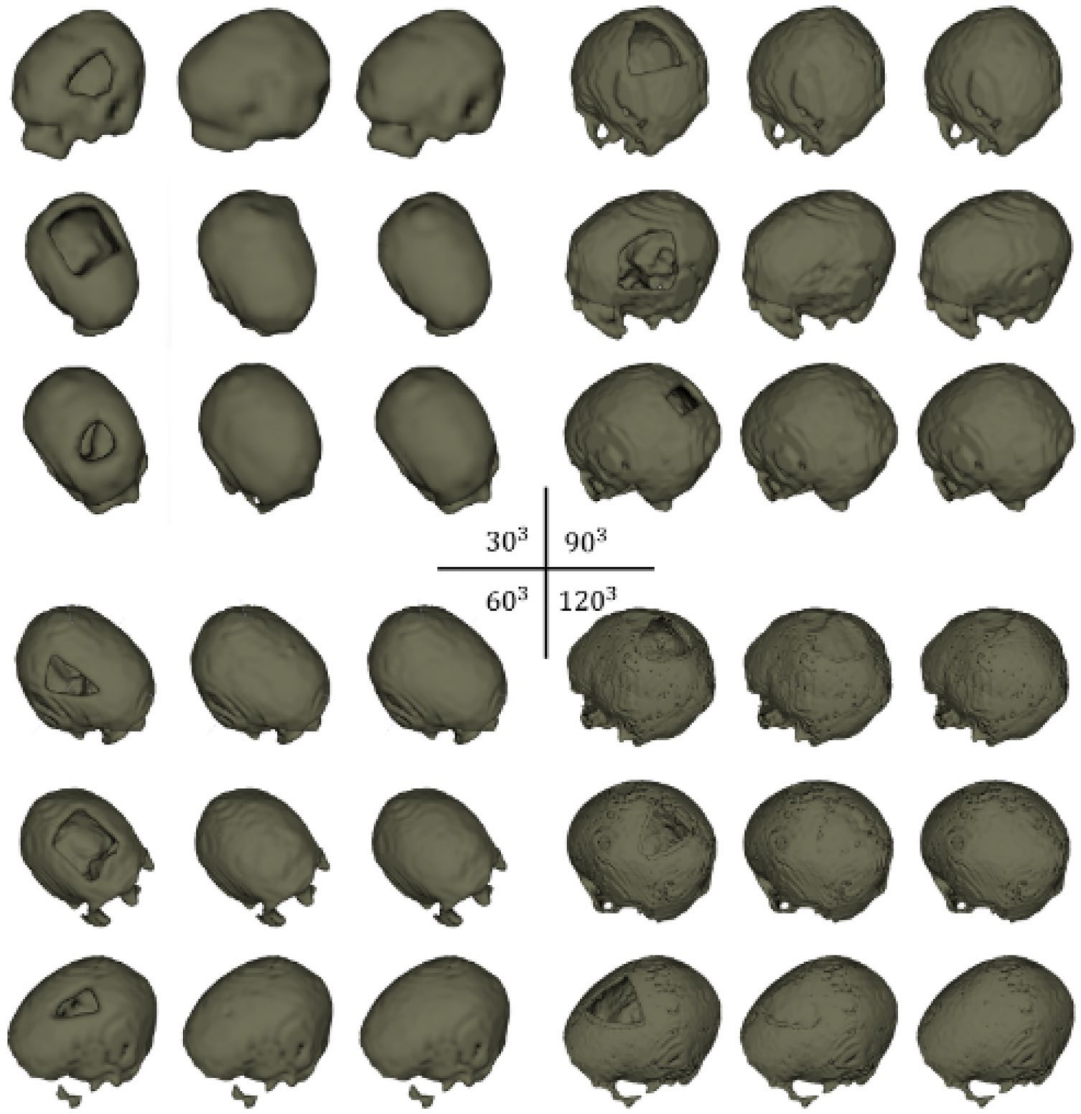
Examples of skull shape completion results with sparse CNN on the MRI skull dataset at different resolutions. The first to third column in each example shows the input defective skull grids, the predictions and the ground truth, respectively.

**Figure 7:**
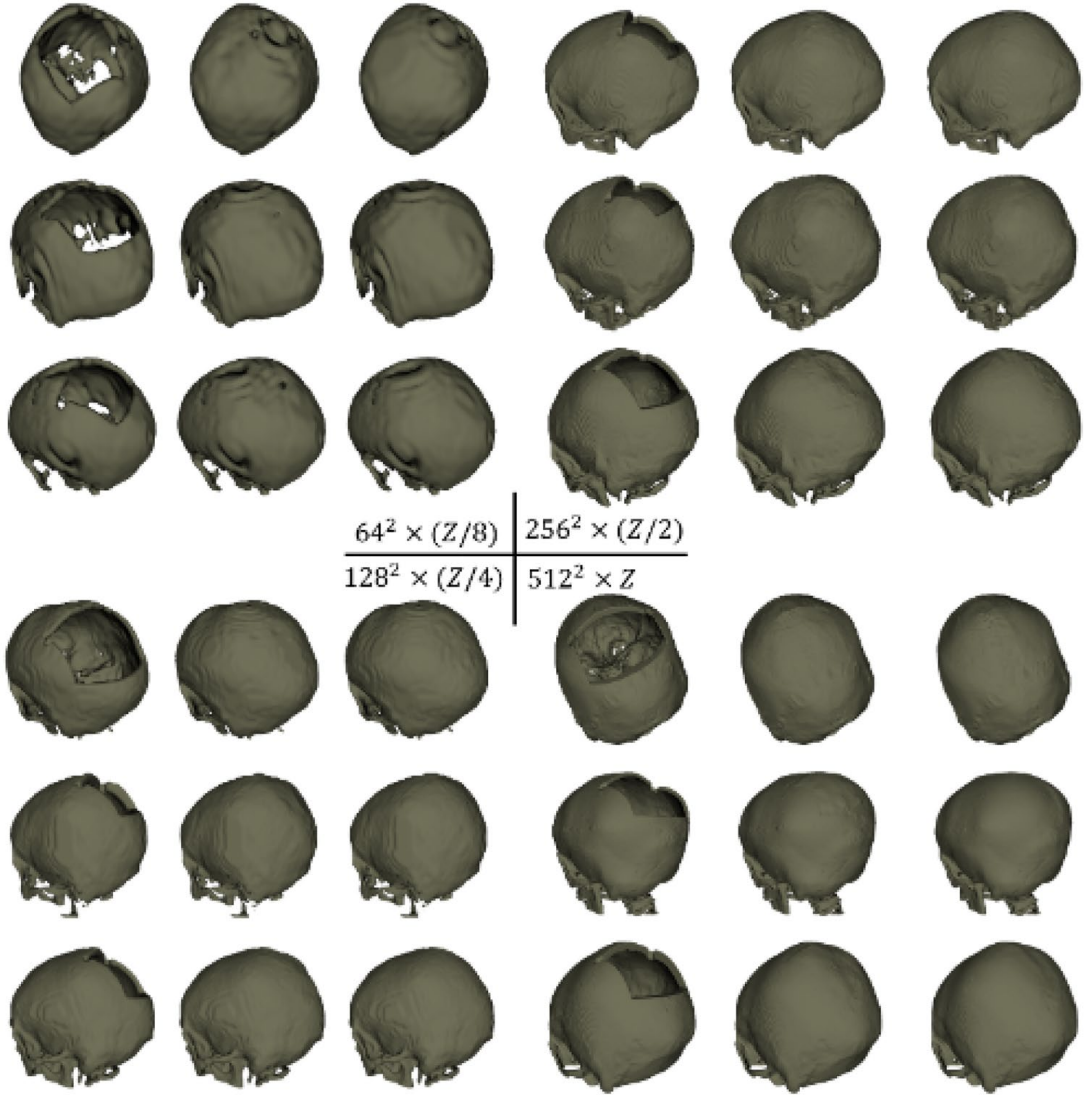
Shape completion results with sparse CNN on the CT skull dataset at different resolutions. The first to third column in each example shows the input defective skull grids, the predictions and the ground truth, respectively.

Figure 5 shows a comparison of the estimated memory consumption of dense and sparse CNN at different image resolutions during training and inference, as well as a comparison of memory consumption of sparse CNN with different batch sizes at image resolution $64^2 \times (Z/8)$ during training. Tables 4 and 5 report the estimated memory usage (in *GB*). With each increase in the image resolution, the image size increases cubically ($\times 8$). During training, the memory consumption of the sparse CNN increases in an approximately linear manner when the image resolution is no more than $256^2 \times (Z/2)$. At $512^2 \times Z$ resolution, the memory usage quadruples ($\times 4$). For the dense CNN, the memory usage demonstrates non-linear growth. During inference, the memory usage of the sparse CNN increases linearly at all resolutions, and the memory consumption of the sparse CNN increases linearly with respect to batch size. Furthermore, the sparse CNN with *ch*2 channels possesses over 40 times the parameters than with *ch*1 channels, whereas the memory increases by less than two times. We take this as indication that, for a sparse CNN, raising the model complexity to improve the prediction accuracy does not cause dramatic increases in memory usage. Figures 6 and 7 show the qualitative completion results on the MRI and CT datasets at different resolutions.

We use the average GPU execution time per skull image to portray the runtime speed of the sparse CNN. For training, we measure the duration of training for 100 iterations and compute the average time per iteration. For inference, we measure the time it takes to run on the entire test set (100 images). Batch size is set to one in both cases. We experimented on the CT data using the shape completion model (*ch*1). At resolution $64^2 \times (Z/8)$, $128^2 \times (Z/4)$ and $256^2 \times (Z/2)$, the training/inference time (*s*) per image is roughly 0.28/0.22, 0.32/0.30 and 0.71/0.54, excluding data loading. We can see that both training and inference time increases linearly with respect to resolutions. Note that the time measured the same way for the dense CNN is not directly comparable to that of the sparse CNN, as the variable i.e., the amount of computational resources they occupy in runtime, can not be controlled during measurement.

**Table 4:** Comparison of estimated memory consumption (the second to last row in *GB*) during training and inference between the sparse and dense CNN at different image resolutions (the first row).

cat. $\backslash$ $I_s$	64	128	256	512
Sparse train	**1.5119**	**1.6256**	**2.7341**	11.3049
Sparse test	**1.4519**	**1.5097**	**1.8905**	2.7993
Dense train	1.6543	1.9043	4.8145	–
Dense test	1.6699	1.8184	2.6934	–

Significant values are in bold.

**Table 5:** Sparse CNN memory consumption (the second and third row in *GB*) with different batch sizes (the first row) at resolution $64^2\times (Z/8)$ during training.

*ch* $\backslash$batch	2	3	4	5	6	7	8	9	10	16	32
*ch*1	1.5119	1.5494	1.5780	1.6164	1.6557	1.6867	1.7151	1.7950	1.8459	2.1180	3.8395
*ch*2	1.9071	–	2.0054	–	–	–	2.3729	2.3232	2.5116	–	–

Keep in mind that, for the sparse CNN, the time and memory growth reported above is not strictly linear, especially during training at high resolutions. The memory and time overhead includes space and computation reserved for voxel coordinates, coordinate mapping and other implementation-related costs.

**Table 6:** Quantitative results - DSC and RE for the skull shape super-resolution task on the CT dataset.

64$\rightarrow$128	64$\Rightarrow$128	64$\rightarrow$256	64 $\Rightarrow$256	64$\rightarrow$512	64 $\Rightarrow$ 512	128 $\rightarrow$ 256	128 $\Rightarrow$ 256	Completion
**0.8750**	0.8359	**0.8779**	0.8359	**0.6640**	0.6402	**0.9372**	0.9146	0.9876
**1.3821**	1.8685	**1.3589**	1.8942	**3.7850**	4.2358	**0.7187**	0.9867	0.1443

The first row shows the initial (the number at the left hand side of an arrow) and target (the number at the right hand side of an arrow) resolutions. The second and last row show the DSC and RE (%), respectively. $\rightarrow$ denotes super-resolution and denotes $\Rightarrow$) interpolation.

Significant values are in bold.

**Figure 8:**
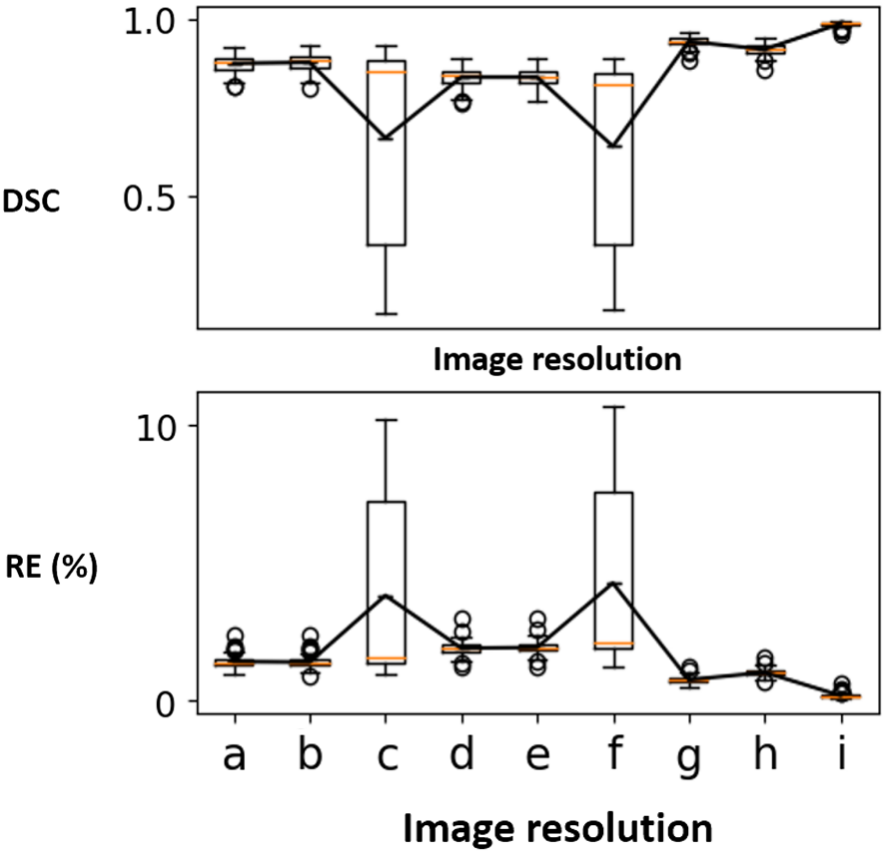
DSC (top) and RE (bottom, %) for the super-resolution task on the CT dataset. Horizontal axis corresponds to the image resolutions. a: $64^2 \times (Z/8) \rightarrow 128^2 \times (Z/4)$ b: $64^2 \times (Z/8) \rightarrow 256^2 \times (Z/2)$ c: $64^2 \times (Z/8) \rightarrow 512^2 \times Z$ d: $64^2 \times (Z/8) \Rightarrow 128^2 \times (Z/4)$ e: $64^2 \times (Z/8) \Rightarrow 256^2 \times (Z/2)$ f: $64^2 \times (Z/8) \Rightarrow 512^2 \times Z$ g: $128^2 \times (Z/4) \rightarrow 256^2 \times (Z/2)$ h: $128^2 \times (Z/4) \Rightarrow 256^2 \times (Z/2)$ i: shape completion at 256. The dash-lined boxes contain the zoomed-in boxplots.

Table 6 shows the quantitative evaluation results for the super-resolution task. In Table 6, $\rightarrow$ represents super-resolution using the sparse CNN, and $\Rightarrow$ represents up-scaling using interpolation. Figure 8 shows the DSC and RE distributions. We can see that super-resolution with a sparse CNN outperforms interpolation-based up-scaling. Besides, super-resolution directly from the lowest-resolution to the highest resolution (i.e., $64^2 \times (Z/8) \rightarrow 512^2 \times Z$) yields the worst results, and the sparse CNN shows better performance at smaller resolution gaps. Table 6 compares super-resolution and shape completion at resolution $256^2 \times (Z/2)$. The results suggest that sparse CNN might be better at the completion task.

**Figure 9:**
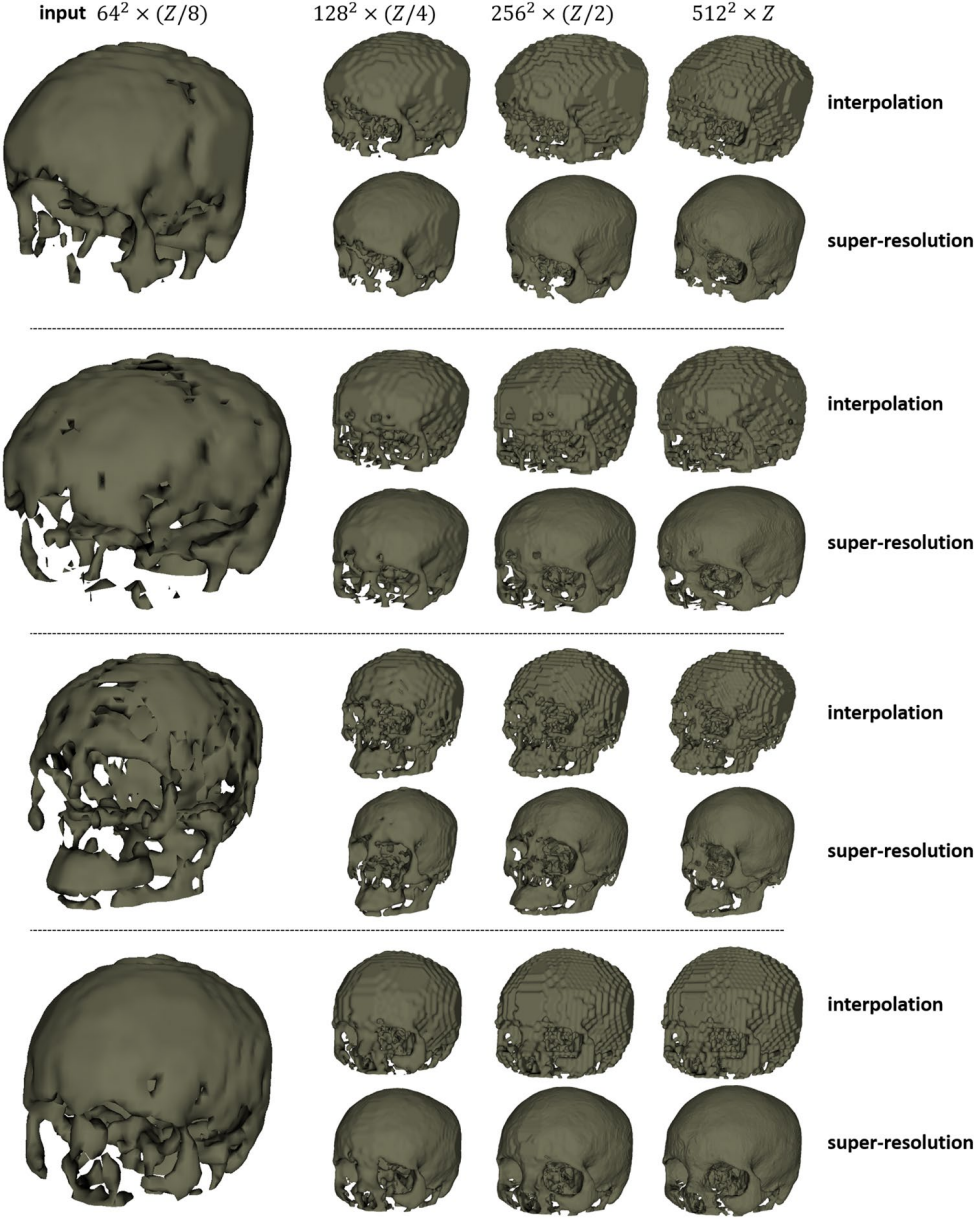
Qualitative results of skull shape super-resolution. The first column shows coarse, completed skulls at resolution $64^2\times (Z/8)$. The second to last column show the interpolation and super-resolution results at resolution $128^2 \times (Z/4)$, $256^2 \times (Z/2)$ and $512^2 \times Z$.

The qualitative results in Fig. 9 further demonstrate the advantages of super-resolution using a sparse CNN. We can see that the missing geometric details in and around the craniofacial area of the coarse skulls can be effectively recovered in the final super-resolution output.

## Discussion, conclusions and future work

In this paper, we have presented a comprehensive evaluation of sparse CNN architectures in two skull reconstruction tasks: skull shape completion and skull shape super-resolution. Results show that a sparse CNN significantly outperforms a traditional dense CNN with respect to speed, quality and memory efficiency on sparse data. Unlike existing dense CNN-based approaches that often compromise accuracy for memory (or vice versa), sparse CNNs are inherently designed for spatially sparse data and are therefore exempt from the accuracy-memory tradeoff, achieving both high accuracy and computation efficiency (e.g., lower memory usage) on the skull reconstruction task. Employing dense CNNs rather than the more advantageous sparse CNNs in early deep learning-based skull reconstruction studies^19,31^ is not out of suitability but convenience, since existing dense CNNs can be straightforwardly applied to voxel grid data as discussed in Section II. One of the limitations of current sparse CNN frameworks, such as the *Minkowski Engine* used in our study, is that the voxel coordinates as well as the associated coordinate management tools need to be created and stored to memorize the spatial relationship of the non-zero voxels during convolutions, causing computation overhead in comparison to dense CNN. Another limitation is that, if not initialized properly, the generative transposed convolutional layers might generate a large amount of points and cause false out-of-memory errors during training. Therefore, one future direction worth investigating is to regularize the generative layers or to use shape priors on the output to prevent the network from generating random amount of points. Additionally, the sparse CNN failed on the out-of-distribution test set of the AutoImplant Challenge, meaning that the network was overfitting to skull defect patterns and lacking generalizability, even if given a full-image context during training. We presume that the network would fail on real craniotomy skulls as well, since craniotomy defects tend to be more irregular than the synthetic defects used in our shape completion experiments. We have yet to decide on the cause of the failure, and future efforts on this issue are still required. In the Supplementary Material, we provided additional experiments and results on other spatially sparse medical images, such as the heart, aortic vessels, trachea and esophagus, in a segmentation task. The results indicate that, with moderate increase of computation and memory, the quality of the initial segmentation masks from a dense CNN can be substantially improved using the proposed sparse CNN model.

### Supplementary Information

Supplementary Information.

## Data Availability

The datasets generated and/or analysed during the current study are available in the Figshare repository https://doi.org/10.6084/m9.figshare.14161307.v1.
