# Supplementary material for: Sparse convolutional neural network for high-resolution skull shape completion and shape super-resolution
Source: Sci Rep. 2023 Nov 19;13:20229. doi: 10.1038/s41598-023-47437-6 (PMC10658170; doi:10.1038/s41598-023-47437-6)
Supplement: Supplementary file 1 — Supplementary Information. [file 41598_2023_47437_MOESM1_ESM.pdf]

Appendices

A. Additional Results on skull shape super-resolution.

Figure A.1 shows a visual comparison of shape completion and super-resolution results at the same resolution level.

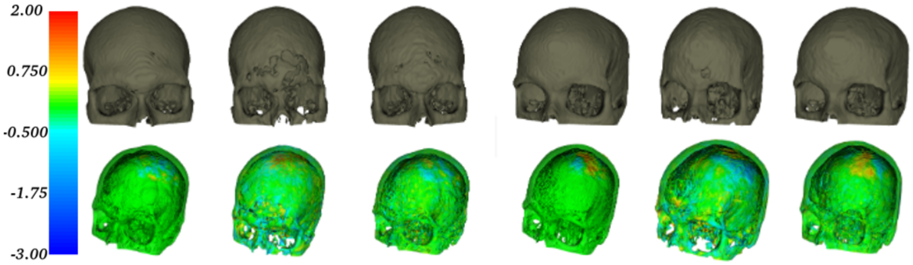

Figure A.1: Comparison of completed skulls at resolution 256. In each example, the first column shows the skull obtained from shape completion at resolution 256. The second and third column show the skull obtained from skull shape super-resolution from 64 and 128. The second row shows the colormap of signed mesh distance between predictions and the ground truth.

B. Implant Generation Results

Figure B.1 shows the generated implants at resolution $512^2 \times Z$ (without any post-processing).

C. Additional Experiments on other Spatially Sparse Medical Images

In this section, additional experiments and results of sparse CNN-based super-resolution on other spatially sparse medical images were provided. The dataset used in the experiments was obtained from the SegTHOR challenge (`https://competitions.codalab.org/competitions/21145`) that addresses the problem of automatic segmentation of organs at risk. The dataset contains 40 CT scans as well as the segmentation masks of the heart (green), aorta (yellow), trachea (blue) and esophagus (red), as can be seen from Figure C.1. The segmentation masks are spatially sparse with very low voxel occupancy rate (VOR), as can be seen from Table C.1. The dataset contains 20 CT scans without the ground truth segmentation masks for evaluation. The CT scans as well as the segmentation masks are of resolution $512 \times 512 \times Z$.

Workflow: Firstly, we downsampled the images to 128^3 and trained a U-Net style dense CNN (1803988 trainable parameters) for automatic segmentation of the organs from the CT scans. Secondly, inference was run on the CT scans in the training and test set to generate the coarse (128^3) segmentation masks. Thirdly, the coarse masks were up-scaled to their original resolution $512 \times 512 \times Z$ via interpolation. Fourthly, we used the up-scaled masks as well as the original ground truth masks from the training

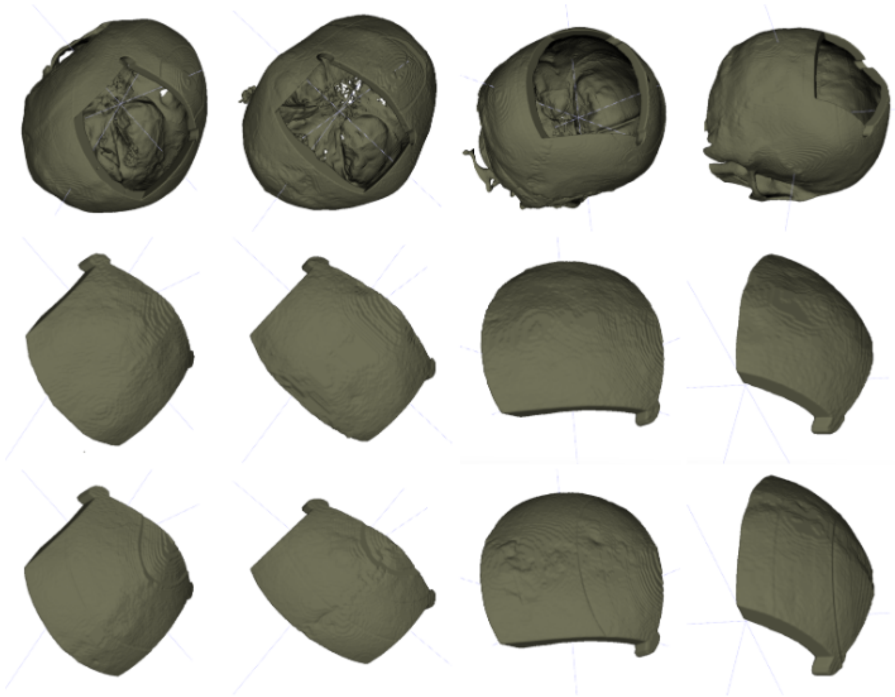

Figure B.1: Implants (second row) obtained by taking the difference between the defective skulls (first row) and the completed skulls at resolution 512. The last row shows the ground truth.

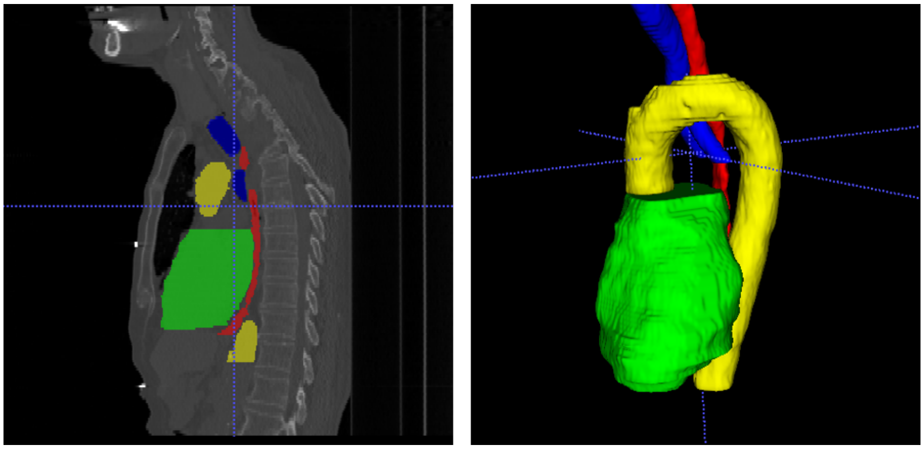

Figure C.1: A CT scan and the ground truth organ segmentation masks of the heart (green), aorta (yellow), trachea (blue) and esophagus (red) from the SegTHOR challenge.

set to train a sparse CNN (the same sparse CNN used for skull super-resolution in the main manuscript) for super-resolution. Lastly, we run the inference of the trained sparse CNN on the up-scaled masks from the test set, to obtain the final high-resolution segmentation masks for these organs.

organ	train	test	VOR (%)
aorta	2.05	1.75	0.20
heart	2.46	2.38	0.79
trachea	1.73	1.64	0.04
esophagus	1.77	1.64	0.05

Table C.1: Voxel occupancy rate (VOR) and the memory usage (in *GB*) during training and inference for different organs.

Figure C.2 - C.5 show the qualitative results of the aorta, heart, esophagus and trachea images. It is worth noting that, as the organs in the dataset are even more sparse than the skulls (Table C.1), training on the full $512 \times 512 \times Z$ resolution for the super-resolution task takes only moderate amount of GPU memory (Table C.1), while the super-resolution step can substantially improve the quality of the segmentation masks, as can be seen from Figure C.2 - Figure C.5. Figure C.6 shows the combined segmentation masks of the organs viewed in 2D and 3D, from sparse CNN.

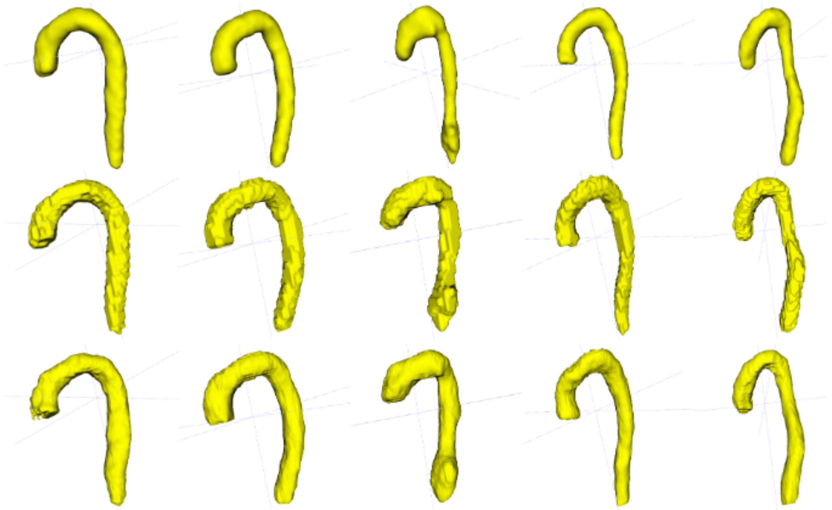

Figure C.2: Super-resolution results of the aorta images. The first to last row shows the coarse aorta mask predictions (128^3) from the dense CNN, the up-scaled aorta masks ($512 \times 512 \times Z$) and the super-resolution output from sparse CNN ($512 \times 512 \times Z$).

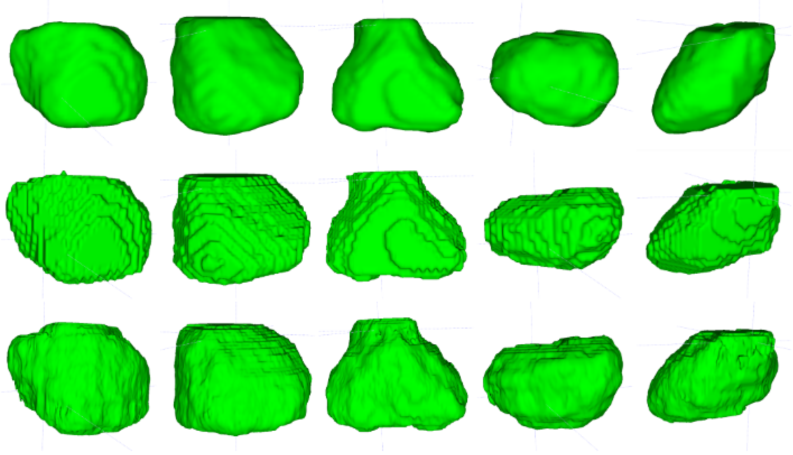

Figure C.3: Super-resolution results of the heart images. The first to last row shows the coarse heart mask predictions (128^3) from the dense CNN, the up-scaled heart masks ($512 \times 512 \times Z$) and the super-resolution output from sparse CNN ($512 \times 512 \times Z$).

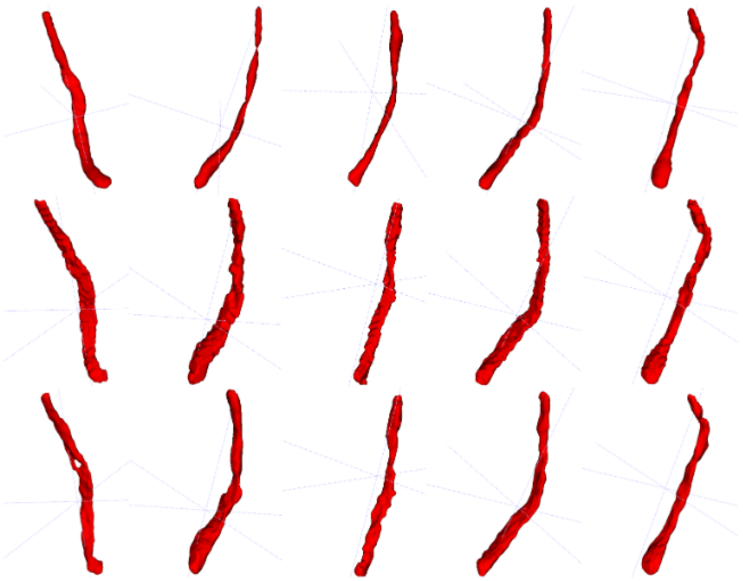

Figure C.4: Super-resolution results of the esophagus images. The first to last row shows the coarse esophagus mask predictions (128^3) from the dense CNN, the up-scaled esophagus masks ($512 \times 512 \times Z$) and the super-resolution output from sparse CNN ($512 \times 512 \times Z$).

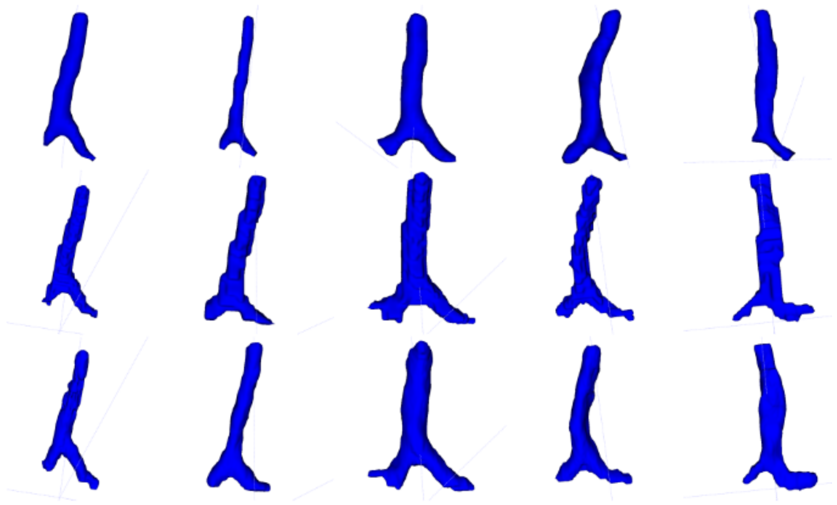

Figure C.5: Super-resolution results of the trachea images. The first to last row shows the coarse trachea mask predictions (128^3) from the dense CNN, the up-scaled trachea masks ($512 \times 512 \times Z$) and the super-resolution output from sparse CNN ($512 \times 512 \times Z$).

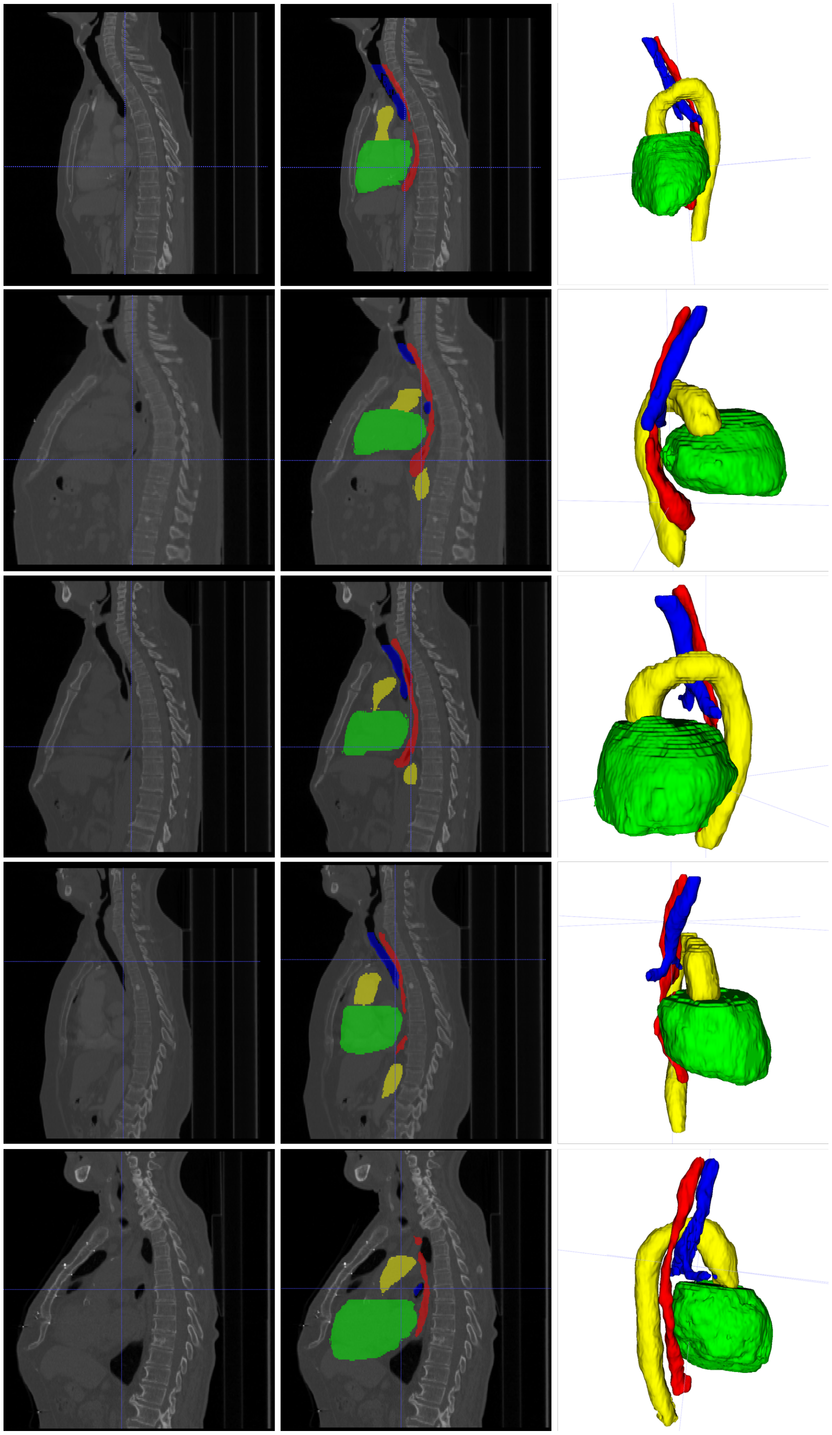

Figure C.6: The segmentation masks of the organs viewed in 2D (second column) and 3D (third column). The first column shows a slice of the CT scan.
